# Respiratory Failure Due to Bilateral Vocal Fold Paralysis Following Laparoscopic Cholecystectomy: A Rare Presentation of Myasthenia Gravis

**DOI:** 10.7759/cureus.97706

**Published:** 2025-11-24

**Authors:** Bruno Santiago, Vitor A Felippe, Gustavo M Sousa, Claudia R Machado, Marcos A Lessa

**Affiliations:** 1 Anesthesia, College of Medical Science, Rio de Janeiro State University, Rio de Janeiro, BRA; 2 Anesthesia, Clementino Fraga Filho University Hospital, Federal University of Rio de Janeiro, Rio de Janeiro, BRA; 3 Anesthesia, Brazilian National Cancer Institute, Rio de Janeiro, BRA; 4 Anesthesia, Faculty of Medical Sciences, Rio de Janeiro State University, Rio de Janeiro, BRA; 5 Anesthesia, University of Iowa Carver College of Medicine, Iowa City, USA

**Keywords:** anesthesia, emergency, myasthenia gravis, perioperative medicine, respiratory failure, stridor

## Abstract

Myasthenia gravis (MG) is an autoimmune disorder affecting the neuromuscular junctions. A 50-year-old woman with well-controlled MG developed stridor and respiratory failure on the first postoperative day after undergoing laparoscopic cholecystectomy with total intravenous anesthesia and sugammadex reversal. During orotracheal intubation, bronchoscopy revealed bilateral vocal fold paralysis in the adducted position. After failed extubation attempts, tracheostomy was required. The patient improved with optimized MG management and was discharged after three weeks. This rare MG manifestation emphasizes the importance of early stridor recognition, difficult airway preparedness, and adherence to American Society of Anesthesiologists guidelines for airway management and bronchoscopy.

## Introduction

Acute postoperative respiratory complications are a major source of morbidity among patients with pre-existing neuromuscular diseases. In these individuals, impaired ventilatory mechanics and upper-airway dysfunction can lead to rapid respiratory decompensation. Myasthenia gravis (MG) - an autoimmune disorder of the neuromuscular junction characterized by fluctuating ocular, bulbar, and respiratory muscle weakness [1] - typically causes respiratory impairment due to weakness of the diaphragm and thoracic pump muscles. However, postoperative laryngeal involvement, although uncommon, may result in stridor and fixed upper-airway obstruction. These manifestations pose diagnostic and therapeutic challenges that demand early recognition, structured decision-making, and a coordinated multidisciplinary approach [2-7].

Reports describing MG with isolated or predominant laryngeal muscle dysfunction are exceedingly rare. Only a few cases in the literature document postoperative presentations marked by acute stridor and upper-airway obstruction [4-6]. The scarcity of such reports highlights the diagnostic difficulty of this atypical presentation, which can easily be misinterpreted as laryngospasm, airway edema, or vocal cord injury. In the perioperative setting, multiple confounding factors - such as residual neuromuscular blockade and airway trauma - further complicate the recognition of laryngeal myasthenic involvement. Accurate diagnosis, therefore, requires a high index of suspicion and multidisciplinary evaluation. This case contributes to the limited body of evidence describing this unusual manifestation of MG.

This case report was approved by the Institutional Ethics Committee of Pedro Ernesto University Hospital/UERJ (approval number: 7.657.338). The manuscript adheres to the relevant EQUATOR guideline and was prepared in accordance with the CARE checklist [8]. Written informed consent for publication was obtained from the patient.

## Case presentation

A 50-year-old woman (body mass index 28 kg/m²) with generalized MG, diagnosed five years earlier and managed with pyridostigmine 60 mg/day, presented for elective laparoscopic cholecystectomy. She had no prior history of respiratory crises or airway involvement. Preoperative evaluation revealed American Society of Anesthesiologists (ASA) [9] Physical Status II and Revised Cardiac Risk Index [10] class II. Routine monitors, quantitative neuromuscular monitoring, and bispectral index were applied, and an 18-gauge peripheral intravenous catheter was placed. The baseline quantitative train-of-four (TOF) ratio prior to muscle relaxation was 1.0, indicating preserved neuromuscular transmission under stable MG control.

Anesthesia management

Induction and maintenance were achieved using target-controlled infusions of propofol (100-150 µg·kg⁻¹·min⁻¹) and remifentanil (0.05-0.2 µg·kg⁻¹·min⁻¹). Rocuronium 25 mg was administered to facilitate tracheal intubation and to ensure adequate muscle relaxation for the laparoscopic procedure, with continuous quantitative neuromuscular monitoring throughout the case. The surgery proceeded uneventfully. At closure, sugammadex 4 mg·kg⁻¹ was administered for deep block reversal (TOF count 0), and the TOF ratio reached 1.0 prior to extubation. The patient was extubated in the operating room and transferred to the ICU for routine observation, given her MG diagnosis.

Postoperative course

On postoperative day 1, while meeting ICU discharge criteria, the patient developed acute inspiratory stridor within minutes of receiving metamizole (dipyrone) 1 g IV. Oxygen saturation fell to 84% despite supplemental oxygen and repositioning. Importantly, she exhibited no rash, bronchospasm, or hemodynamic instability, reducing the likelihood of anaphylaxis. Given the simultaneous concern for ventilation and intubation difficulty, the anesthesia team proceeded with awake fiberoptic intubation following preoxygenation and topical anesthesia using the spray-as-you-go technique.

Diagnostic findings

**Figure 1:**
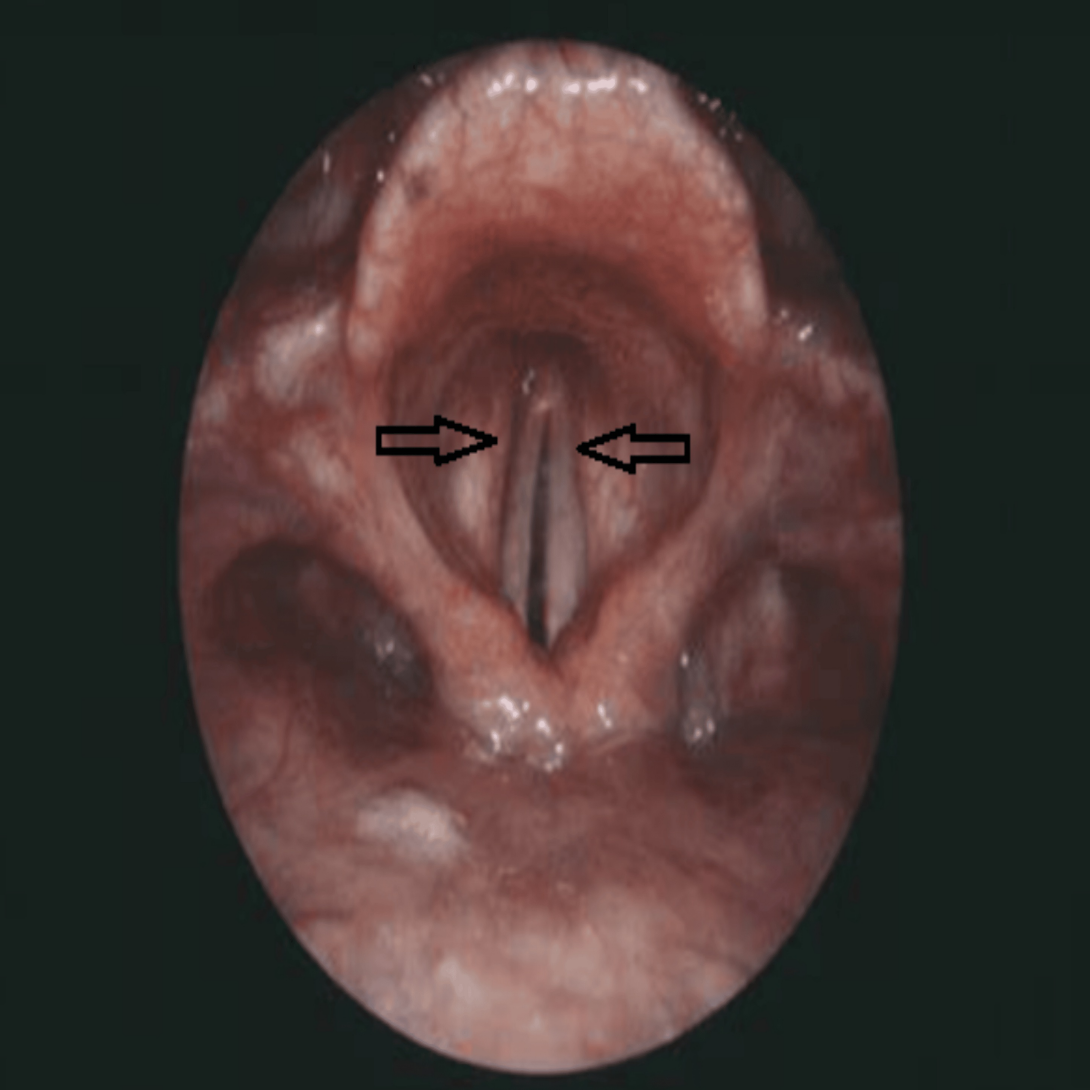
Fiberoptic view of the larynx demonstrating bilateral vocal fold paralysis. Both vocal folds are fixed in the adducted position (arrows), resulting in a markedly narrowed glottic aperture. No evidence of edema, bleeding, or structural trauma is observed. The image was obtained during diagnostic bronchoscopy performed shortly after onset of postoperative inspiratory stridor, confirming the diagnosis of bilateral vocal fold paralysis in a patient with myasthenia gravis.

Fiberoptic examination revealed both vocal folds fixed in the adducted position during orotracheal intubation, without edema, bleeding, or evidence of traumatic injury (Figure 1), consistent with bilateral vocal fold paralysis. This finding, together with the absence of allergic or structural causes, supported a diagnosis of MG-related laryngeal weakness presenting as postoperative airway obstruction.

Management and outcome

The patient was treated with intravenous dexamethasone 10 mg and optimization of anticholinesterase therapy under neurology supervision. Corticosteroid therapy was initiated to reduce inflammation and enhance neuromuscular transmission. Given the localized presentation and progressive improvement, escalation to immunoglobulin therapy or plasmapheresis was deemed unnecessary.

A trial of extubation was attempted after 48 hours of ventilatory support once the patient met the following criteria: adequate spontaneous breathing effort, stable hemodynamics, tidal volume >5 mL/kg, vital capacity >15 mL/kg, negative inspiratory force <−25 cmH₂O, TOF ≥0.9, and effective cough reflex. The trial was conducted under pressure support 8 cmH₂O, PEEP 5 cmH₂O, and FiO₂ <0.4, maintaining SpO₂ >94%. Despite meeting objective criteria, she developed recurrent severe stridor shortly after extubation, necessitating reintubation.

Tracheostomy and weaning: Given the persistence of upper airway obstruction, a tracheostomy was performed under general anesthesia, with the patient sedated using fentanyl (2 µg·kg⁻¹), dexmedetomidine (0.5-0.7 µg·kg⁻¹·h⁻¹), and propofol (50-100 µg·kg⁻¹·min⁻¹), and no neuromuscular blocking agents were administered. This approach aimed to maintain spontaneous ventilation and minimize the risk of prolonged weakness in a patient with MG.

Postoperatively, sedation was maintained in the ICU with dexmedetomidine (0.2-0.4 µg·kg⁻¹·h⁻¹) for comfort and ventilatory synchrony while preserving respiratory drive. Weaning from mechanical ventilation was achieved gradually through daily spontaneous breathing trials, followed by progressive capping and downsizing of the tracheostomy tube. Once she demonstrated stable gas exchange, adequate cough, and absence of stridor, she was discharged home breathing spontaneously through a downsized tracheostomy tube, with outpatient follow-up planned for decannulation after recovery of vocal fold mobility.

## Discussion

MG is an autoimmune disorder caused by autoantibodies directed against acetylcholine receptors (AChR) on the postsynaptic membrane, resulting in impaired neuromuscular transmission and fluctuating muscle weakness. Its global prevalence is estimated at 150-250 cases per million people, with incidence peaks in young women and in men over 60 years of age [1].

Clinically, MG presents with fatigable muscle weakness that worsens with exertion and improves with rest. Diagnosis is suggested by a transient response to the edrophonium test, a decremental pattern on nerve conduction studies, and elevated serum levels of anti-AChR or anti-muscle-specific tyrosine kinase (MuSK) antibodies. Notably, up to 20% of patients with generalized MG have undetectable anti-AChR antibodies [1].

Ocular, bulbar, and respiratory muscles are commonly affected [4]. Although respiratory impairment is well recognized in MG, it usually results from weakness of the diaphragm and thoracic musculature rather than upper airway obstruction. The present case, bilateral vocal fold paralysis in the postoperative period, represents a rare and clinically significant manifestation. Its recognition broadens current understanding of atypical MG presentations, particularly those triggered by perioperative stress. With the growing number of elective surgical procedures, more patients with neuromuscular disorders are being exposed to anesthesia and surgery, increasing the risk of MG exacerbations [3].

Upper airway obstruction in MG remains poorly understood. Laryngeal involvement can cause weakness of both vocal fold adduction and abduction, leading to a breathy or whispering voice, reduced pitch range, and phonatory fatigue. As adductor weakness progresses, the vocal folds move toward the midline, producing stridor and dyspnea. Bilateral vocal fold paralysis in the adducted position represents a severe and uncommon form of upper airway obstruction. The differential diagnosis includes traumatic injury to the recurrent laryngeal nerve, post-extubation edema, laryngospasm, allergic reactions, and infectious causes. Distinguishing among these etiologies is essential for timely and targeted management. Table 1 summarizes previously reported cases of laryngeal stridor as a presenting feature of MG.

**Table 1:** Reported cases in the literature of laryngeal stridor as a presenting feature of myasthenia gravis (MG). Summary of previously published case reports describing laryngeal stridor as the initial or predominant manifestation of MG. Data include patient demographics, type of study, presenting features, diagnostic confirmation, and outcomes. Abbreviations: AChR = acetylcholine receptor; IVIg = intravenous immunoglobulin; MG = myasthenia gravis.

Author/Year	Type of Study	Presentation	Outcome/Key Findings
Colp et al., 1980 [3]	Case report	Female, 44 y; postoperative stridor and acute respiratory failure due to vocal cord paralysis.	Developed generalized muscle weakness requiring tracheostomy and ventilatory support. Edrophonium test confirmed MG; improved on pyridostigmine 15 mg q6h; discharged after two weeks.
Foulks et al., 1981 [11]	Case report	Male, 25 y; dyspnea and stridor after exposure to hydrochloric gas; bilateral vocal cord paralysis on laryngoscopy.	MG diagnosed with edrophonium test; treated with pyridostigmine, prednisone, and thymectomy with good recovery.
Schmidt-Nowara et al., 1984 [12]	Case report	Male, 63 y; choking, severe dyspnea, apnea, cyanosis, and stridor.	Diagnosis confirmed by edrophonium; treated with pyridostigmine 60 mg/day; improved clinically with addition of extended-release pyridostigmine for nocturnal symptoms.
Fairley and Hughes, 1992 [13]	Case report	Male, 46 y; stridor, dysphagia, nasal regurgitation, dysarthria, diplopia, ptosis; fiberoptic exam showed paralysis of vocal fold abductors.	Edrophonium test positive; improved with pyridostigmine 60 mg × 5/day and thymectomy.
Job et al., 1992 [14]	Case report	Female, 55 y; recent-onset stridor and dysphagia; vocal fold paralysis in paramedian position; diplopia and ophthalmoplegia.	Developed respiratory failure requiring tracheostomy; MG confirmed with edrophonium; died of sepsis.
Hanson et al., 1996 [2]	Case report	Female, 72 y; stridor and respiratory distress; dysphagia and masticatory muscle fatigue.	Extrathoracic obstruction on spirometry; improved after thymectomy and titration of pyridostigmine and prednisone.
Abul Matin et al., 1999 [15]	Case report	Female, 16 y; sudden-onset inspiratory stridor; paradoxical vocal fold movement.	MG confirmed by edrophonium; partial improvement with pyridostigmine and immunosuppressive therapy proposed.
Osei-Lah et al., 1999 [16]	Case report	Male, 61 y; stridor and respiratory effort; bilateral vocal fold paralysis in paramedian position; diplopia and cervical weakness.	MG with thymoma confirmed by edrophonium and MRI; treated with pyridostigmine and thymectomy; later relapse with respiratory failure and death.
Hara et al., 2007 [17]	Case report	Male, 56 y; weakness, fatigue of masticatory muscles, diplopia; worsening stridor at end of day; vocal folds in paramedian position.	MG confirmed by decremental response and anti-MuSK antibodies; partial improvement with corticosteroids; required tracheostomy.
Sethi et al., 2011 [18]	Case report	Male, 68 y; acute respiratory distress and stridor; vocal fold paralysis in paramedian position; eyelid ptosis.	Edrophonium test positive; decremental response on nerve conduction; improved with neostigmine 3 mg q6h.
Balabbigari et al., 2020 [19]	Case report	Female, 51 y; dysphagia and hoarseness progressing to stridor and respiratory failure; multiple failed extubations.	MG confirmed by high AChR antibody titers; treated with corticosteroids and plasmapheresis; stabilized on outpatient immunosuppressive therapy.
Arman et al., 2021 [20]	Case report	Male, 53 y; intermittent stridor; initially diagnosed with idiopathic bilateral vocal fold paralysis.	MG confirmed later by EMG and antibodies; improved after IVIg and mycophenolate; recovery of vocal fold movement after eight months.

In this case, awake fiberoptic intubation was appropriately chosen due to the combined risk of anatomical and physiological airway difficulty. This approach is formally endorsed by the ASA for patients at risk of failed ventilation and intubation and/or aspiration [7]. The technique preserves spontaneous ventilation and airway reflexes, minimizing the risk of a “cannot ventilate, cannot intubate” scenario. The “spray-as-you-go” method for topical anesthesia, combined with titrated conscious sedation, optimizes patient comfort while maintaining airway safety. However, successful awake intubation requires meticulous preparation, experienced personnel, and judicious drug selection, as oversedation or inadequate topicalization can precipitate airway obstruction.

Pre-anesthetic evaluation in MG patients is vital for identifying risk factors and developing an individualized perioperative plan to prevent postoperative airway failure and reintubation [2]. These patients demonstrate increased sensitivity to neuromuscular blocking agents (NMBAs), which can produce exaggerated or unpredictable effects. Quantitative neuromuscular monitoring and tailored reversal are therefore essential. In this case, rocuronium provided appropriate muscle relaxation for laparoscopic surgery, and its reversal with sugammadex was confirmed with quantitative monitoring. Nevertheless, further research is needed to define optimal dosing strategies, given the variability of NMBA sensitivity in MG.

The temporal association between intravenous metamizole administration and the onset of respiratory distress is intriguing but remains speculative. Although metamizole is generally safe, idiosyncratic reactions cannot be excluded, and no robust data link it directly to MG exacerbations. Some commercial dipyrone formulations contain magnesium salts, which are known inhibitors of neuromuscular transmission. Magnesium reduces acetylcholine release at the presynaptic terminal and potentiates the effects of both depolarizing and nondepolarizing NMBAs. Thus, in MG patients, exposure to magnesium-containing dipyrone preparations could theoretically worsen muscle weakness or precipitate respiratory failure, especially in the postoperative period when respiratory reserve is reduced. This observation highlights the importance of reviewing medication excipients and maintaining pharmacovigilance in this population. From a pharmacologic standpoint, this association underscores the need for cautious perioperative drug selection in MG, as patients with this condition are particularly sensitive to medications that impair neuromuscular transmission. Although a causal link cannot be established, heightened awareness and further pharmacovigilance studies are warranted.

One of the major challenges in this area is the lack of robust clinical evidence directly linking MG to bilateral laryngeal paralysis. Current knowledge is largely based on isolated case reports and small series, which, although informative, limit the establishment of standardized diagnostic and therapeutic approaches. Consequently, causality between dipyrone administration and respiratory failure cannot be confirmed. Multifactorial mechanisms-including underlying disease progression, anesthetic agents, and surgical stress-should also be considered. Long-term follow-up data on vocal fold recovery and airway outcomes remain limited in the literature. Including such data in future reports would help clarify prognosis and highlight the importance of multidisciplinary follow-up in patients with laryngeal MG.

This evidentiary gap underscores the need for prospective, multicenter studies or registries to better define risk factors, optimize perioperative management, and strengthen the evidence base for clinical decision-making in complex neuromuscular cases. Addressing these research gaps will enhance understanding of postoperative airway complications in MG and contribute to developing preventive strategies.

## Conclusions

This case underscores a rare but clinically significant postoperative manifestation of MG-bilateral vocal fold paralysis causing acute upper airway obstruction. It highlights the importance of vigilant postoperative monitoring, the use of quantitative neuromuscular monitoring with appropriate reversal to ensure safe extubation, and early recognition of stridor with preparedness for difficult airway management. Heightened awareness of this atypical presentation can aid timely diagnosis and intervention, improving safety and outcomes in patients with neuromuscular disorders undergoing surgery.
